# Scar incision and endoscopic balloon dilation for severe benign esophageal strictures

**DOI:** 10.1016/j.vgie.2025.08.009

**Published:** 2025-09-02

**Authors:** Naoya Tada, Akira Dobashi, Mamoru Ito, Toshiki Futakuchi, Masakuni Kobayashi, Naoto Tamai, Kazuki Sumiyama

**Affiliations:** Department of Endoscopy, The Jikei University School of Medicine, Tokyo, Japan

## Abstract

**Background and Aims:**

Endoscopic balloon dilatation (EBD) is the primary treatment for benign esophageal strictures after endoscopic or surgical resection of esophageal cancer. Resolving these strictures often requires multiple sessions over time and risks perforation. To address these challenges, we developed a combined technique integrating scar incision with EBD, wherein scar incisions are created using a needle-knife before balloon dilation.

**Methods:**

Linear incisions were made at the stricture site using a needle-knife, targeting areas with pronounced fibrosis or requiring tension release. Subsequently, EBD was performed to dilate the entire stricture site. These procedures were performed in 1 session. Additional EBD or EBD with scar incision sessions were conducted at intervals until patients became asymptomatic.

**Results:**

We evaluated EBD with scar incision in 5 patients with severe esophageal strictures after endoscopic or surgical resection for esophageal cancer. All patients became asymptomatic within 3 months. One patient experienced restricture 139 days after the final EBD but improved after an additional EBD session. No adverse events requiring intervention occurred.

**Conclusions:**

EBD with scar incision effectively achieves asymptomatic resolution within a short period. Pre-creating incisions with a needle-knife enhances the efficacy and safety of balloon dilation.

## Introduction

The management of esophageal strictures after endoscopic resection or surgery comes with significant challenges. Moreover, the indications for endoscopic submucosal dissection (ESD) of the esophagus have expanded in recent years, increasing post-ESD strictures.^1^ In certain cases, esophageal strictures post-ESD are characterized by an extended stricture length, complicating short-term resolution. Multiple sessions of endoscopic balloon dilation (EBD) are often necessary to achieve resolution of a stricture.^2^, ^3^, ^4^ In addition, the application of high pressure by a balloon in cases of severe fibrosis can lead to the sudden rupture of all layers, with perforation rates of 0.3% to 1.1%.^2^^,^^5^ Consequently, we have developed a dilation method for esophageal strictures to enhance the effectiveness and safety of balloon dilation. This technique involves creating several linear incisions at the site of severe fibrosis using a needle-knife followed by balloon dilation (EBD with scar incision). Herein, we demonstrate this method in 5 patients (Table 1).

**Table 1:** Patient characteristics and outcomes of EBD with scar incision

Variables	Case 1	Case 2	Case 3	Case 4	Case 5
Age, y	77	76	44	65	79
Sex	Male	Male	Female	Male	Male
Location	Ut	Mt	Ut	Mt	Ce
Cause of the stricture	Surgery	ESD	ESD	ESD	ESD
Number of conventional EBDs before initial EBD with scar incision	12	6	11	1	5
Duration from initial EBD to initial EBD with scar incision, d	293	68	139	11	86
Asymptomatic status at 3 months after initial EBD with scar incision	Yes	Yes	Yes	Yes	Yes
Number of EBD with scar incision sessions, n	1	2	1	2	1
Duration from initial EBD with scar incision to target diameter, d	0	71	11	85	36
Procedure time, min					
First EBD with scar incision	26.0	22.0	25.5	26.0	10.0
Second EBD with scar incision	–	22.5	–	17.0	–
Number of conventional EBDs after initial EBD with scar incision	0	4	1	6	3
Adverse events related to EBD with scar incision or EBD	No	No	No	No	No
Restricture after achieving asymptomatic status	No	No	No	No	Yes
Observational period after final EBD or EBD with scar incision session, mo	12	56	69	42	57

*Ce*, Cervical esophagus; *EBD*, endoscopic balloon dilation; *ESD*, endoscopic submucosal dissection; *Mt*, middle thoracic esophagus; *Ut*, upper thoracic esophagus; –, not available.

## EBD with scar incision

**Figure 1:**
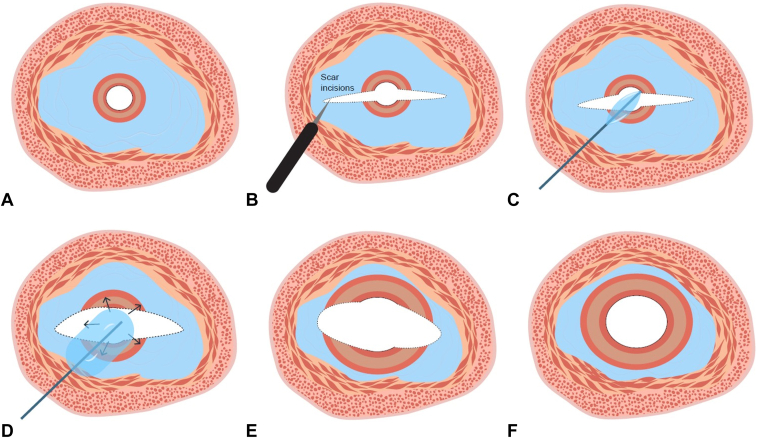
The schematic image of the endoscopic balloon dilation (EBD) with scar incision. **A,** Benign esophageal stricture. **B,** Scar incisions. **C,** Inserting the balloon. **D,** Balloon dilation. **E,** After the EBD with scar incision. **F,** Achieving the resolution of the stricture.

A single incision or multiple linear incisions were created at the target areas of the stricture to alleviate fibrosis-related tension after ESD or surgery using an ESD needle-knife (DualKnife J; Olympus, Tokyo, Japan; or FlushKnife; Fujifilm, Tokyo, Japan). The electrosurgical unit used was VIO 300 D or VIO 3 (Erbe Elektromedizin GmbH, Tübingen, Germany), with settings determined by the endoscopist (eg, VIO 3: ENDO CUT I, effect 2, duration 2, interval 3; swiftCOAG, effect 5). To ensure a safe incision, the procedure was performed as follows: Longitudinal mucosubmucosal incisions for severe scarring were made with a 1.5-mm needle-knife. Submucosal injection was omitted because it is not feasible in such fibrotic regions. The knife was manipulated with care taken that it was not perpendicular to the incision surface, sliding it from the proximal to the distal side. Without making deep incision, balloon dilation was performed once a sufficient portion of the fibrotic tissue had been incised to create an adequate tear at the incision site. The dilation diameter was determined based on the diameter applied in the previous session, following the “rule of 3” with each dilation limited to an increase of no more than 3 mm.^6^ Direct visualization through the balloon was used to avoid excessive dilation, leading to perforation. It would be preferable that the muscular layer becomes visible after EBD (Fig. 1). Target diameters differed because postoperative anastomotic strictures have lower perforation risk than post-ESD or other strictures, allowing larger balloon dilation.^7^ This technique should not be performed for long strictures when the knife tip cannot be visualized, because blind manipulation compromises safety. Procedural steps remain unchanged regardless of previous local steroid injection after ESD (Video 1, available online at www.videogie.org).

The procedure was performed on an inpatient basis. After the procedure, patients were permitted clear fluids on the same day and advanced to oral intake on the following day. Discharge was typically on the second day postprocedure.

After the initial EBD with scar incision, conventional EBD or EBD with scar incision was repeated at appropriate intervals of several weeks to 1 month, depending on the clinical course, until dysphagia was resolved, as assessed by the dysphagia score commonly used in Japan Clinical Oncology Group studies.^8^ Asymptomatic status was defined as achieving the target diameter, which required both a dysphagia score of 0 and the successful passage of an endoscope with an outer diameter of ≥8.9 mm through the stricture, followed by a 3-month period without the need for EBD. Patients were informed of the potential adverse events associated with this procedure, similar to EBD, including the risks of perforation and bleeding. The study was approved by the Ethics Committee of The Jikei University School of Medicine (approval 36-436, March 10, 2025).

### Case 1

**Figure 2:**
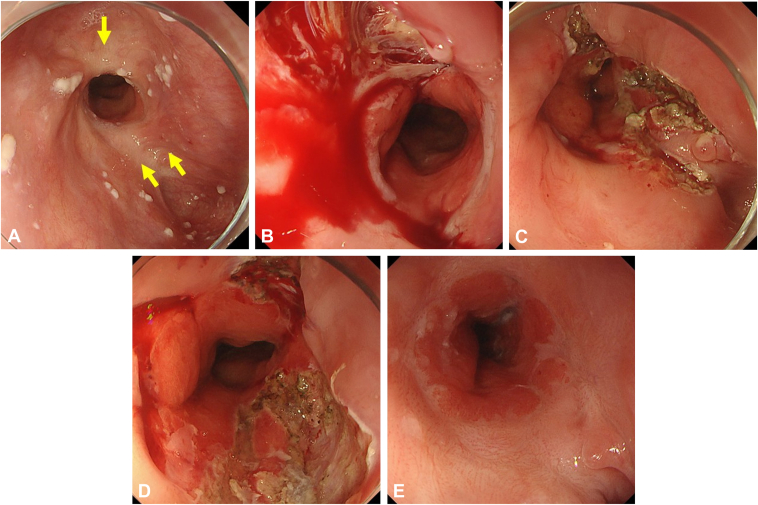
Case 1. **A,** Stricture at anastomosis site after esophageal surgery. *Yellow arrows* note fibrotic scars targeted for incision. **B,** The tear created by a conventional EBD session occurred in the same area between the 9- and 10-o'clock positions. **C,** Three linear scar incisions. **D,** After EBD following scar incisions. **E,** Achieving asymptomatic status without additional conventional EBD sessions. *EBD*, Endoscopic balloon dilation.

A 77-year-old man underwent EBD for a stricture at the esophagogastric anastomosis of upper thoracic esophagus after surgery for esophageal cancer (Fig. 2). The initial EBD commenced at 12 mm, and over the course of 12 EBD sessions, the balloon diameter was gradually increased to 20 mm. In each EBD session, a tear was made by dilation at the 10-o'clock position of the stricture, where severe fibrosis was not observed, and the area of the severe scar remained unchanged. Therefore, severely fibrotic areas at the 4-o'clock and 12-o'clock positions were linearly incised, and EBD was performed up to a size of 20 mm. After EBD with scar incision, 20 mg of triamcinolone acetonide was locally injected into the normal epithelium surrounding the incision site. An asymptomatic status was achieved 11 days after EBD with scar incision, without the need for an additional EBD session. No restricture was imposed during the 12-month observation period.

### Case 2

**Figure 3:**
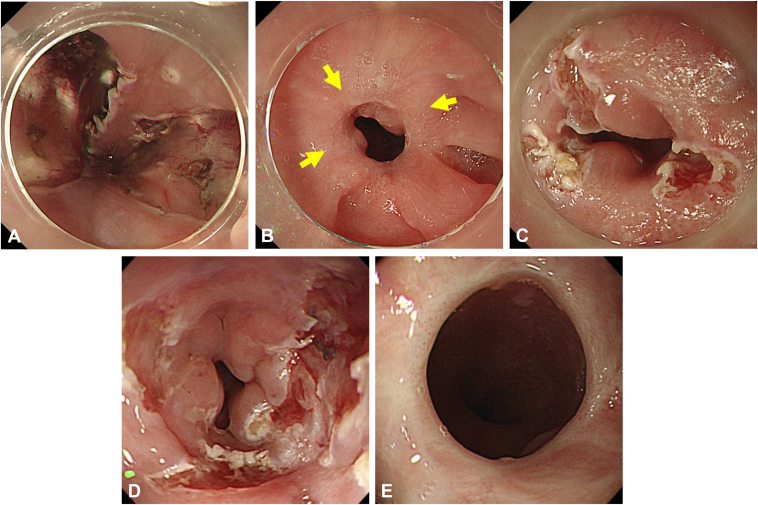
Case 2. **A,** Two mucosal defects occupied over three-quarters of the circumference. Injected triamcinolone acetonide in the submucosa of the defect. **B,** The stricture before EBD with scar incision. *Yellow arrows* note the fibrotic scars targeted for incision. **C,** Three linear scar incisions. **D,** After EBD following scar incisions. **E,** Asymptomatic status achieved 71 days after the initial EBD with scar incision. *EBD*, Endoscopic balloon dilation.

A 76-year-old man underwent esophageal ESD of the middle thoracic esophagus for 2 esophageal cancers resulting in a stricture. Six EBD sessions were performed over a 3-month period, using a balloon with a maximum diameter of 15 mm. However, the stricture remained unresolved (Fig. 3). Because of the patient's advanced age and difficulty with frequent hospital visits, EBD with scar incision was performed to facilitate early resolution. During the first EBD with scar incision, 3 linear scar incisions were made, and EBD was performed up to 12-mm dilation. After 2 conventional EBDs, a second EBDs with scar incision was conducted with a 15-mm balloon. Two EBDs with scar incision sessions and 4 additional conventional EBDs were subsequently conducted, with the patient achieving asymptomatic status 71 days after the initial EBD with scar incision. No restricture was imposed during the 56-month observation period after final conventional EBD.

### Case 3

**Figure 4:**
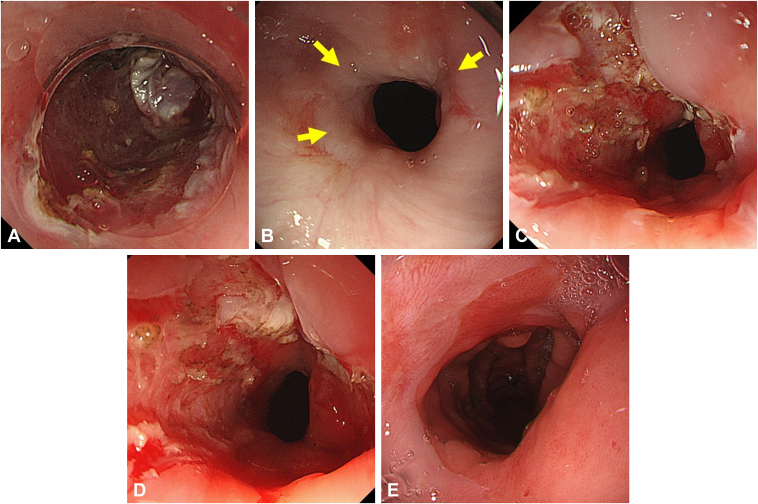
Case 3. **A,** After esophageal endoscopic submucosal dissection near anastomosis following esophageal surgery. **B,** Stricture before EBD with scar incision. *Yellow arrows* note the fibrotic scars targeted for incision. **C,** Three linear scar incisions. **D,** After EBD following scar incisions. **E,** Asymptomatic status achieved 11 days after the initial EBD with scar incision. *EBD*, Endoscopic balloon dilation.

A 44-year-old woman had a history of esophagectomy with gastric conduit reconstruction for advanced esophageal cancer. A new superficial esophageal cancer developed involving the anastomotic site. ESD was performed, after which a stricture occurred in the upper thoracic esophagus (Fig. 4). Despite 11 EBD sessions, 14-mm dilation briefly allowed scope passage, but a stricture quickly recurred. Consequently, EBD with scar incision was performed involving 3 linear incisions followed by balloon dilatation to 15 mm. An additional EBD up to 16.5 mm was performed 11 days after EBD with scar incision, but the endoscope could pass through the stricture site. No stricture recurrence was observed during the 69-month observation period after final conventional EBD.

### Case 4

**Figure 5:**
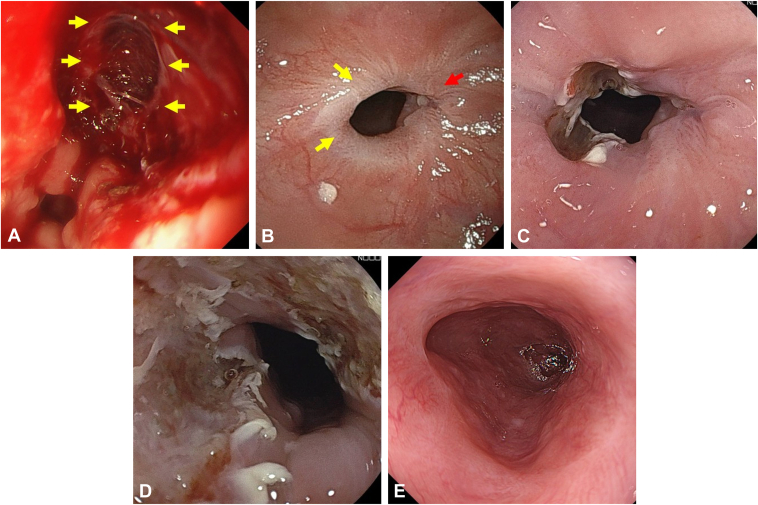
Case 4. **A,** Perforation occurred at the 1- to 2-o'clock positions during a conventional EBD session for a stricture following ESD (*yellow arrows*). **B,** Stricture before EBD with scar incision. The scar due to perforation existing at 2- to 3-o'clock positions (*red arrow*). *Yellow arrows* note the fibrotic scars targeted for incision. **C,** Two linear scar incisions are created at the 7- and 11-o'clock positions, avoiding the postperforation side. **D,** After EBD following scar incisions. **E,** Asymptomatic status achieved 85 days after the initial EBD with scar incision. *EBD*, Endoscopic balloon dilation; *ESD*, endoscopic submucosal dissection.

A 65-year-old man underwent circumferential esophageal ESD for 2 lesions in close proximity in the middle thoracic esophagus, resulting in a severe stricture. Perforation occurred during the initial EBD using a balloon with a diameter of 15 mm (Fig. 5). Stent placement for a benign GI stricture or perforation is not covered by insurance in Japan. Therefore, the patient was conservatively managed by fasting, intravenous fluids, and antibiotic therapy. The condition resolved in 2 weeks. As repeat conventional EBD posed a risk of perforation at the same site, EBD with scar incision was performed after the perforation site healed 16 days following the initial EBD. Two linear incisions were made on the opposite side of the perforation, followed by EBD. The incisions created using a needle-knife absorbed the pressure from the EBD, preventing further perforation at the same site. Two EBDs with scar incision sessions and 6 EBD sessions were conducted, considering the need for caution against perforation, with gradual increase up to a 12-mm balloon. The stricture was resolved within 3 months without reperforation.

### Case 5

**Figure 6:**
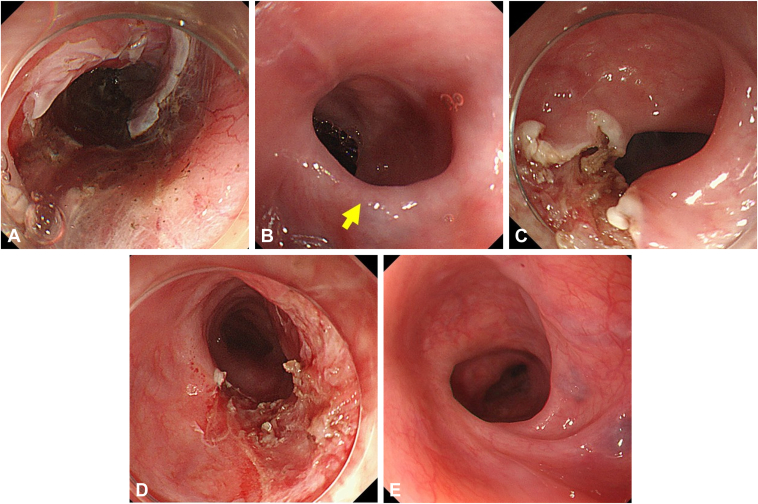
Case 5. **A,** Three-quarter mucosal defect after esophageal endoscopic submucosal dissection. **B,** Stricture before EBD with scar incision. *Yellow arrows* note the fibrotic scars targeted for incision. **C,** One linear scar incision. **D,** After EBD following scar incisions. **E,** Asymptomatic status achieved 36 days after the initial EBD with scar incision. *EBD*, Endoscopic balloon dilation.

A 79-year-old man underwent 5 EBD sessions using a balloon with a maximum diameter of 15 mm for a stricture caused by esophageal ESD in the cervical esophagus (Fig. 6). However, EBD failed to create effective tears in the stricture due to pressure dispersion to nonfibrotic areas, preventing passage of the endoscope through the stricture site. Consequently, EBD with scar incision was performed to alleviate tension at the site of severe fibrosis by incisions. After EBD with scar incision, 2 additional EBD sessions were conducted to treat the stricture. Restricture occurred 139 days after the initial EBD with scar incision, requiring an additional 15-mm EBD. No further restrictures were observed during the 57-month observation period after final conventional EBD.

## Discussion

Our study demonstrates that EBD with scar incision is a safe and effective technique for managing various esophageal stricture scenarios after esophageal ESD or surgery. Asymptomatic status was achieved in all cases within 3 months and maintained, except in 1 case. The restricture in case 5 was effectively managed with a single additional EBD.

The radial incision and cutting method has been reported for refractory strictures, but it often necessitates frequent prophylactic EBDs until the treated site is covered by scarring (median, 4 times).^9^ The restricture rates ranged from 58.7% to 92%, with perforation rates of 3.5% to 4.0%.^9^, ^10^, ^11^ This may be attributed to radial incision and cutting removing the circumferential epithelium, akin to circumferential ESD. In contrast, EBD with scar incision can selectively incise severe fibrosis in the mucosubmucosa while preserving portions of the healthy epithelium, which may serve as a foundation for tissue regeneration and aid in preventing further stricture formation.

In EBD, mucosubmucosal tears often occur abruptly during balloon dilation within areas of high tension caused by stiff fibrosis and scars. The depth of these tears cannot be controlled, heightening the risk of perforation. Reducing the tension of severe fibrosis through incisions before balloon dilation may enhance the efficacy and safety of EBD.

Selective incision of areas with high tension is a meaningful approach for treating strictures because relieving mechanical tension suppresses fibrosis and scar formation.^12^ This represents a significant advantage of EBD with scar incision, which cannot be achieved through conventional EBD, because EBD cannot target specific areas for tearing. When EBD is performed on a patient who previously experienced perforation, it is necessary to select a smaller balloon size to prevent reperforation by gradually increasing the balloon size. However, tears from EBD may still occur in weaker tissues; therefore, the risk of reperforation persists. In contrast, creating selective incisions before balloon dilation can guide tears to specific locations, enabling the creation of tears in areas other than the previous perforation site. This strategy may help avoid reperforation.

Although stent placement is an alternative option for the treatment of refractory strictures, it carries significant risks, such as difficulties with reintervention or retrieval in cases of migration. The present technique is an extension of EBD that allows repeated sessions when necessary. Considering its lower cost and risk profile than stent placement, this approach may be more practical and should be considered before it.

However, this method has some limitations. First, this technique may reduce the number of EBD sessions required to achieve symptom relief; yet, symptom improvement is not guaranteed after a single session. Further research is required to determine the optimal indications for this method. Second, EBD with scar incision incurs higher costs due to the use of a needle-knife and requires more procedural time compared to EBD alone, thereby necessitating selective application. On the basis of our experience, suitable indications include cases not improved by repeated EBD, repeated tears at the same site, failed tear creation, or perforation. However, introducing EBD with scar incision to reduce the number of EBD sessions required to achieve an asymptomatic status could potentially reduce the overall cost. Third, the technical aspects of making linear incisions should be discussed, although endoscopists find ESD relatively straightforward to perform. For operators unfamiliar with needle-knife manipulation, there may be an increased risk of perforation. The appropriate incision depth warrants further consideration. In our experience, exposing the muscle layer through incisions is unnecessary, as subsequent EBD could also induce tear fibrosis at the incision sites. The efficacy of steroid injections after EBD with scar incision remains unclear. However, we postulate that steroid injections will enhance outcomes. Scar incision was performed exclusively using a needle-knife. The efficacy of other devices, such as an endoscopic knife with hood attachment (eg, ITknife2; Olympus, Tokyo, Japan), warrants further investigation.

In conclusion, EBD with scar incision is a feasible method to enhance the effectiveness and safety of EBD, representing a promising new approach for cases with inadequate response to EBD or with high risk of perforation in conventional EBD. Further research will clarify its role in the managing of esophageal strictures.

## Patient consent

The patients in this article have given written informed consent to publication of their case details.

## Disclosure

All authors disclosed no financial relationships.

## Supplementary data

Video 1 Scar incision and endoscopic balloon dilation for severe benign esophageal strictures.
